# Case of an Extra-Uterine Fœtus

**Published:** 1787

**Authors:** Michael Underwood

**Affiliations:** Licentiate in Midwifery of the Royal College of Physicians, and Physician to the British Lying-in Hospital in London


### LONDON MEDICAL JOURNAL,

FOR THE YEAR 1787,

### PART THE FOURTH.

I. *Case of an Extra-uterine Fœtus. Communicated in a Letter to Dr.* Simmons *by* Michael Underwood, *M. D. Licentiate in Midwifery, of the Royal College of Physicians, and Physician to the British Lying-in Hospital in London.*

MRS. Sheppard, of Snow Hill, London, naturally a healthy woman, rather under the middle ſize, muſcular, but not inclined to be corpulent, was married in 1731, being then in her twenty-third year. She ſoon became pregnant, and miſcarried at the end of ten weeks. She after this miſcarried five or ſix times at nearly the ſame period of geſtation.

In 1738, when in her thirtieth year, ſhe again proved with child, and went on well till ſhe had quickened. Unfortunately, at the end of five months, being violently frightened, ſhe fainted away, and, upon her recovery, felt

ſomething (as ſhe expreſſed it) break within her, and from that period was for a conſiderable time ſubject to returns of the fainting. She continued, however, to increaſe in bulk, and at the end of nine months, being affected with the grinding pains of labour, ſhe ſent for a midwife, who, though ſhe could not diſcover any opening of the os uteri, was fully perſuaded that the abdominal tumor was owing to an enlargement of the womb. The pains continued to increaſe next day, but without producing any viſible change in the os uteri. Dr. Bamber and other phyſicians being conſulted, internal medicines and clyſters were exhibited; notwithſtanding all which, ſhe continued in racking torture for four days, when ſhe fell aſleep, and, ſoon after awaked eaſy. During the following night ſhe was affected with repeated faintings, and milk was then found to be in her breaſts. She continued for a ſhort time to be tolerably eaſy, but ſoon had ſome returns of pain, and, for the firſt time, perceived a black, fœtid, bloody diſcharge from the vagina, which laſted four or five days, and during the five ſucceeding weeks ſhe had repeated appearances of this kind, attended tended at times with violent pains, and a difcharge of coagula, refembling pieces of flefh. The fwelling of the abdomen began gradually to diminifh after the firft difcharge, and at the time the patient got abroad (which was two years after) was reduced to half the former fize, and continued diminifhing for the three fucceeding years; during all which time fhe had painful difcharges at irregular periods, and paffed feveral of thefe folid coagula, which the byeftanders imagined (contrary to the opinion of the medical gentlemen) to be parts of a placenta.

After thefe five years fhe paffed no more folid coagula, but had the catamenia regularly, though painfully, and difcoloured, for about two years more. In her thirty-feventh year, viz. in 1745, fhe thought herfelf breeding again, as fhe increafed gradually in bulk, as before, to what fhe thought her full term of nine months, when, being feized with labour pains, which continued regularly for a whole day together, her midwife pronounced her to be certainly with child, but without any appearance of natural labour.

She continued to be haraffed with grinding pains, equally ineffectual, and frequently attended with ſome diſcharge, every fortnight or three weeks, for about two years; after which ſhe was attended by the late Sir William Watſon, who continued to viſit her occaſionally the five ſucceeding years, during all which time the enlargement of the abdomen remained, and the pains frequently returned. He procured her temporary relief by opiates and clyſters; but her complaints always recurring, ſhe conſulted the late Dr. Ward, who gave her repeatedly half of one of his ſweating powders, which at firſt relieved her, but after the fourth doſe brought on violent pain of four hours continuance; after which ſhe fell aſleep, and when ſhe awaked was free of pain. In a week afterwards ſhe found herſelf better, her abdomen gradually ſubſiding and her breathing becoming eaſier. The menſes now returned more regularly, and in greater quantity, and in ſix months ſhe was reduced to her natural ſize. She had, nevertheleſs, her uſual and violent pains at times for about thirteen years.

About a year after the ſwelling of the abdomen had diſappeared, ſhe menſtruated more ſparingly, and at longer intervals, and began again to feel an increaſe of the abdomen, which continued continued for near nine months, and then gradually diſappeared.

She had, after this, three more enlargements of the abdomen, of a ſhorter continuance, during the above thirteen years, but had no milk in her breaſts, as in the two former of three and ſeven years.

At the expiration of theſe thirteen years from the ſecond ſuppoſed pregnancy, after ſuffering pains for ſeveral days, ſhe was ſeized, while ſitting on the cloſeſtool, with one more violent than uſual, and paſſed ſomething with great difficulty by the anus, which was found to be the rib of a fœtus. This was in the year 1759, about twenty-one years from her fright during pregnancy. The menſes had then left her about twelve months. From this time ſome bones came away every two or three days for ſeveral weeks, but with more eaſe than the firſt, and ſhe was able in about five weeks to walk about the houſe, but could not for three years walk half a mile. During all this time ſome bones came away every two or three weeks; but after that time ſhe remained eaſy for a quarter or half a year, without parting with any, and then gradually recovered a conſiderable degree of ſtrength.

The

The bones ſhe voided ſeemed to be thoſe of a fœtus of about five months growth, and were thoſe of the ribs, ſcapula, and vertebræ, all of which were paſſed previouſly to the beginning of the year 1770, when I firſt ſaw her, and received from her the above narrative. At this time ſome bones were coming away every three or four days, but with leſs pain than formerly, and I found her, upon the whole, in tolerable good health. During the early part of the year 1771 ſhe voided but few, but towards the cloſe of it paſſed near twenty pieces of bone with conſiderable pain, and ſhe never could walk to any diſtance without ſuffering by it. After this ſhe voided very little bone till towards the end of the year following, when, falling down ſtairs, ſhe bruiſed the os coccygis, which occaſioned pain every time ſhe went to ſtool. Before this fall ſhe had got free of thoſe bearing-down pains which ſhe had been ſo long ſubject to; but after that ſhe had more conſtant pain, though not ſo violent. Several more pieces of bone were paſſed about this period.

During the ſpace of two years after this fall ſhe continued to void pieces of bone with much leſs trouble, and had frequent intervals of eaſe for months together, which enabled her to recover her ſtrength in a great meaſure, though ſhe never was ſo eaſy as before the accident.

Towards the latter end of the year 1774 ſhe was become pretty eaſy, and, by computation, it was found ſhe had paſſed, in the laſt fifteen years, about three hundred ſmall pieces of bone, and half as many larger, which laſt were very thin. At the beginning of 1775 ſhe brought on a painful diſorder of her bowels by an advertiſed purging pill, and after this ſhe paſſed ſeveral pieces of bone, and particularly one, which ſeemed to be an exfoliation of the ileum, near two inches long.

In the courſe of the next year, 1776, many ſmall bones were voided; but after this ſhe remained upwards of a year without paſſing any, and again recovered her health and ſtrength in great degree.

After this ſhe no longer paſſed any large pieces of bone, but ſometimes ſmaller ones, without any other trouble, however, than that of ſome uneaſineſs when ſhe allowed herſelf to become coſtive.

In 1778, when ſhe had arrived at the age of ſeventy years, ſhe received a conſiderable acceſſion of fortune, which (owing probably to a frame enervated by forty years ſuffering) ſo

ſo changed her temper and deranged her mind, that ſhe became peeviſh, emaciated, reſtleſs, and very ſoon after maniacal. She continued in that ſtate till her death, which happened not long ago; and having been removed into the country, when ſhe loſt her ſenſes, there was no opportunity of examining the body.

*Great Marlborough Street,*

June 7th, 1787.

